# Hyaluronan-Metal Gold Nanoparticle Hybrids for Targeted Tumor Cell Therapy

**DOI:** 10.3390/ijms21093085

**Published:** 2020-04-27

**Authors:** Vanessa Sanfilippo, Viviana Carmela Linda Caruso, Lorena Maria Cucci, Rosanna Inturri, Susanna Vaccaro, Cristina Satriano

**Affiliations:** 1 Nano-Hybrid-BioInterfacesLab (NHBIL), Department of Chemical Sciences, University of Catania, Viale Andrea Doria 6, 95125 Catania, Italy; sanfilippo.vanessa@studium.unict.it (V.S.); vcaruso@unict.it (V.C.L.C.); lorena.cucci@unict.it (L.M.C.); 2 Fidia Farmaceutici S.p.A., R&D Unità locale Fidia Research sud, Contrada Pizzuta, 96017 Noto (SR), Italy; rinturri@fidiapharma.it (R.I.); svaccaro@fidiapharma.it (S.V.)

**Keywords:** theranostics, CD44, hyaluronic acid, neuroblastoma cells, prostate cells, nanotoxicity, plasmonics, confocal microscopy

## Abstract

In this study, a novel multifunctional nanoplatform based on core-shell nanoparticles of spherical gold nanoparticles (AuNPs) capped with low and high molecular weight (200 and 700 kDa) hyaluronic acid (HA), was assembled via a green, one-pot redox synthesis method at room temperature. A multitechnique characterization approach by UV-visible spectroscopy, dynamic light scattering and atomic force microscopy pointed to the effective ‘surface decoration’ of the gold nanoparticles by HA, resulting in different grafting densities of the biopolymer chains at the surface of the metal nanoparticle, which in turn affected the physicochemical properties of the nanoparticles. Specifically, the spectral features of the gold plasmonic peak (and the related calculated optical size), the hydrodynamic diameter and the nanoparticle stability were found to depend on the molecular weight of the HA. The CD44-targeting capability of HA-functionalized gold nanoparticles was tested in terms of antibacterial activity and cytotoxicity. An enhanced inhibitory activity against both Gram-negative *Escherichia coli* and Gram-positive *Staphylococcus aureus* was found, with a HA molecular weight (MW)-dependent trend for the HA-capped AuNPs compared to the bare, glucose-capped AuNPs. Cell viability assays performed on two CD44-positive cell models, namely normal human umbilical vein endothelial (HUVEC) and prostate tumor (PC-3) cells, in comparison with neuroblastoma cells (SH-SY5Y), which do not express the CD44 receptor, demonstrated an increased cytotoxicity in neuroblastoma compared to prostate cancer cells upon the cellular treatments by HA–AuNP compared to the bare AuNP, but a receptor-dependent perturbation effect on cytoskeleton actin and lysosomal organelles, as detected by confocal microscopy. These results highlighted the promising potentialities of the HA-decorated gold nanoparticles for selective cytotoxicity in cancer therapy. Confocal microscopy imaging of the two human tumor cell models demonstrated a membrane-confined uptake of HA-capped AuNP in the cancer cells that express CD44 receptors and the different perturbation effects related to molecular weight of HA wrapping the metallic core of the plasmonic nanoparticles on cellular organelles and membrane mobility.

## 1. Introduction

Rapid advances and emerging technologies in nanoscale systems, particularly nanoparticles (NPs), are having a profound impact on tumor diagnosis, treatment and monitoring, because of their ability to detect cancer cells, to provide chemotherapeutic agents and to control the treatment response [1]. Nanometer-sized drug delivery systems offer many advantages compared to traditional medicines, including an increased specificity of the therapy by targeting capability that avoids adverse effects on non-target tissues [2] and the ability to cross the biological barriers that they may encounter by moving towards the target tissue or organ [3]. The latter is easily achieved by the enhanced permeability and retention effect (EPR), as a consequence of the abnormal and leaky vasculature of the tumor environment that permits the tumor-specific accumulation of the nano-sized structures (20–100 nm) with respect to the healthy tissue [4,5].

In recent years, several new-generation drug delivery vehicles, contrast agents and diagnostic systems based on the use of nanoparticles have been developed for theranostic (i.e., therapy + imaging) applications [6]. Examples include PEGylated liposomal doxorubicin, albumin-bound paclitaxel and liposomal irinotecan approved in the USA for the treatment of refractory ovarian carcinoma and Kaposi’s sarcoma, and metastatic breast cancer and pancreatic cancer, respectively [7].

Among the various classes of theranostic nanomaterials, metal nanoparticles and in particular gold nanoparticles (AuNPs) have been widely exploited in nanomedicine, due to their tuneable optical properties, originating from the surface plasmon resonance which gives rise to an intense adsorption band within the visible region for the gold nanospheres and in the near-infrared (NIR) biological window for gold nanorods [8] as well as a relatively low cytotoxicity dependent on the dose, shape, size and type of capping agents used [9]. To this respect, spherical citrated-capped AuNPs in the size range of 10–50 nm have been considered as non-toxic towards several human cell lines [10,11,12,13]. In addition, their easy synthesis [14] and functionalization with a variety of biomolecular ligands [15] as well as no susceptibility to the photobleaching or chemical/thermal denaturation mostly associated with common dyes [16], make them suitable for application in biochemical sensing, medical diagnostics and therapeutic strategies [17,18,19]. To be noted, PEGylated gold nanoparticles have been approved by the Food and Drug Administration for photothermal cancer therapy [20]. Along with the optoelectronic properties, AuNPs show also intrinsic biological features that make them promising for obtaining multifunctional hybrid nanoplatforms. Gold nanoparticles exhibit antioxidant [21,22] and anti-angiogenic properties [23,24] by binding to the heparin-binding growth factors, thus blocking the VEGF-induced angiogenesis [22] which further allows for their applications as anticancer agents [17,23].

In particular, surface modifications with natural biomolecules and polymers are largely used to improve the targeting and the biocompatibility of AuNPs and to achieve specific binding sites on cell-surface receptors [25,26]. Such a strategy can also prevent the spontaneous adsorption of plasma proteins on the AuNP’s surface which may affect the chemical properties of the nanosystems and their interaction with cells [27].

Hyaluronic acid, or hyaluronan, is a linear polysaccharide composed of repeating units of D-glucuronic acid and N-acetyl-d-glucosamine, linked together through alternating β-1,4 and β-1,3 glycosidic bonds [28,29]. Hyaluronic acid (HA) possesses excellent properties of water solubility, biocompatibility, biodegradation, non-toxicity and non-immunogenicity [30]. As a pericellular matrix, it may have effects on ion flux which are important in cellular signaling through membrane ion channels. On the other hand, HA plays also an important role in certain pathologies, including cancer. An increase in HA synthesis has indeed been considered in several malignant tumors, such as colon carcinoma and breast cancer [31,32]. HA promotes the adhesion and migration of tumor cells [33] and forms a protective barrier around them, thus allowing their survival [34].

Different molecular weights of hyaluronic acid can be related to opposite biological effects [35]. For instance, HA oligomers are able to inhibit tumor proliferation in vivo [36], to induce apoptosis [37] and revert resistance to chemotherapy [38]. Low molecular weight (MW) HA is associated with pathological conditions and above all with cancer [39] and can be inducer of inflammation and angiogenesis [40]. Low/intermediate MW HA (20–450 kDa) is known to promote gene expression in macrophages, endothelial cells, eosinophils and certain epithelial cells [41]. Finally, high-MW HA (>1000 kDa) is found in physiological conditions in the extracellular matrix [42] and exhibits anti-angiogenic and immunosuppressive functions [40].

HA is also known to play a role in promoting cell proliferation, differentiation and migration by binding with cells with specific interactions [43]. The biological behavior of HA is mediated by the interaction with proteins, known as hyaladherins, that act as cellular receptors for HA [44].

Based on current knowledge, different HA-binding proteins have been identified and, among them, CD44, a transmembrane glycoprotein receptor that is expressed on endothelial cells in situ and in vitro, is one of the most well-characterized receptors [45]. CD44 is considered to be the primary HA receptor on most cell types including leucocytes, fibroblasts, keratinocytes and epithelial and endothelial cells and it is involved in different cellular processes such as cell adhesion, migration, proliferation and activation as well as HA degradation and uptake [46,47]. Since the discovery that the receptor is overexpressed in a variety of solid tumors, such as pancreatic, breast and lung cancer, many studies have focused on methods for targeting CD44 in an attempt to improve drug delivery and discrimination between healthy and malignant tissue, while reducing residual toxicity [44]. Experimental evidence shows that CD44 is the most effective membrane protein to bind hyaluronic acid, thus HA has been extensively used in developing nanocarriers that demonstrate preferential tumor accumulation and increased cell uptake in cancer cells [44].

The biological response to the HA-CD44 interaction is highly dependent on the HA chain length. Thus, high-MW HA are capable of binding to cell surface receptors by multivalent interactions inducing cell quiescent and showing anti-angiogenic and immunosuppressive [40] responses. On the other hand, low-MW HA interacts monovalently, it can induce inflammatory cytokine release and initiate a series of events that begin with matrix adherence modifications and continue with the activation of other molecules (such as growth factors), degradation of the matrix itself, vessel permeation, extravasation and angiogenesis [40].

In normal cell lines, such as HUVEC (human umbilical vein endothelial cells), the receptor CD44 is also expressed and it is able to link HA; in fact, data reported in literature proved that antibody anti-CD44 inhibited the HA adhesion [45]. The link interaction between hyaluronic acid and the CD44 receptor adhesion molecule can initiate a series of events that begins with matrix adherence modifications and continues with the activation of intracellular signaling pathway which leads to trigger other molecules (such as growth factors), degradation of the matrix itself, angiogenesis, vessel permeation, extravasation [44,48]. All these steps are necessary in the initiation of the metastatic pathway [49,50]. The CD44 receptor is involved in eliciting an innate immune response, through different ways [51]. The strong upregulation of CD44 on leukocytes is related to its role in extravasation of these immune cells from the bloodstream into inflamed tissues and represents an important component of the immune response, that is the major effector of normal resolution of early bacterial infections, especially in a previously immunized setting [52].

The CD44 can be induced in T cells upon monocyte activation by inflammatory stimuli; moreover, an upregulation of the CD44 gene was observed during infections, supporting its possible involvement in both pathogenesis and recovery processes [26,48,52]. The robust upregulation of the CD44 gene during infections supports its involvement in both pathogenesis and recovery [52]. In inflammation, HA may also have a moderating effect through a free radical scavenging [53,54] antioxidant effect [55], as well as through exclusion of tissue-degrading enzymes from the immediate cellular environment and from other structural components of the extracellular matrix [53]. In infection, HA is associated with reduction of bacterial growth and adhesion inhibition on different cell lines; it acts as a physical barrier, inhibiting the epithelial disruption. Moreover, HA acts in vitro as mask for CD44 receptor, inhibiting the *Escherichia coli* adhesion to the receptor [56]. Furthermore, CD44 is known to mediate important aspects of the infection of macrophages with *Staphylococcus aureus* [57].

Since the discovery that the receptor is overexpressed in a variety of solid tumors, such as pancreatic, breast and lung cancer, many studies have focused on methods for targeting CD44 in an attempt to improve drug delivery and discriminate between healthy and malignant tissue, while reducing residual toxicity [44]. One of the earliest pieces of evidence for effective delivery by HA-modified carriers to tumor cells was demonstrated by Eliaz et al. [58]. Successively, several approaches to nanoparticle formulations have been developed that take advantage of the CD44-targeting properties of HA, including the chemical conjugation of HA to pre-formed lipid-based nanocarriers, for the active targeting of small or large active molecules for the treatment of cancer [59], and self-assembling nanosystems for targeted siRNA delivery [60]. Furthermore, nanoparticles modified with HA have been demonstrated to exert better preferential tumor accumulation and increased cell uptake in cancer cells due to a HA–CD44 specific interaction [30,44].

Kumar et al. obtained gold nanoparticles using the extract of eggplant as a reducing agent, while low molecular weight (12 kDa) HA served as a capping and targeting agent [61]. After loading with a drug (metformin) they found a higher apoptotic behavior in CD44 positive-HepG2 cells than in than in CD44 negative-NIH 3T3 cells [61]. In another report, a HA-AuNP complex was prepared by chemical binding of thiolated HA and physical binding of interferon alpha, used for the clinical treatment of hepatitis C virus infection [62]. The AuNPs functionalized with HA can deliver compounds that are intrinsically susceptible to enzymatic degradation or those that exhibit poor intracellular penetration (e.g., siRNA) [63]. Moreover, the conjugation of HA-AuNPs with inhibitor of apoptosis protein-2 specific-RNA (IAP-2 siRNA) led to silencing of the IAP-2 expression, the decreasing of cell proliferation and the triggering of pronounced cell apoptosis; thus, HA-AuNPs could be considered as a promising tool for the therapy of lung cancer [26].

In the present work, hyaluronic acid-gold nanoparticle (HA-AuNP) hybrids were synthetized by reducing the precursor metallic salt with glucose and hyaluronan, acting both as reducing and stabilizing agents. The effective functionalization of the gold colloidal with the polysaccharide and the stability of the nanosystems was inspected by UV-visible (UV-vis) spectroscopy by following the variations of the plasmon peak during the aging time and by dynamic light scattering (DLS) by studying the increasing of the hydrodynamic diameter of the AuNPs upon the HA conjugation.

Bacterial experiments were performed to examine the HA-AuNPs cytotoxic activity towards Gram-negative *Escherichia coli* and Gram-positive *Staphyolococcus aureus*, investigating the inhibitory activity using broth microdilution method. Cellular experiments were performed to test their interactions with human model cell lines, one that does not-express CD44, i.e., CD44-negative neuroblastoma tumor cells, SH-SY5Y [64] and two CD44-positive cell lines, which express the CD44 receptor on their surface membrane, namely prostate cancer, PC-3 [65] and HUVEC [45]. The obtained results pointed out a promising potential of the synthetized hybrids for selective targeting and cytotoxicity in cancer therapy.

## 2. Results

The physicochemical characterization of the HA-coated AuNPs was performed by a multitechnique approach with UV-visible spectroscopy, dynamic light scattering (DLS) and atomic force microscopy (AFM), to scrutinize the plasmonic peak features, the hydrodynamic size and the structure of the core (metal gold)-shell (glycoside) nanoparticles, respectively.

**Figure 1:**
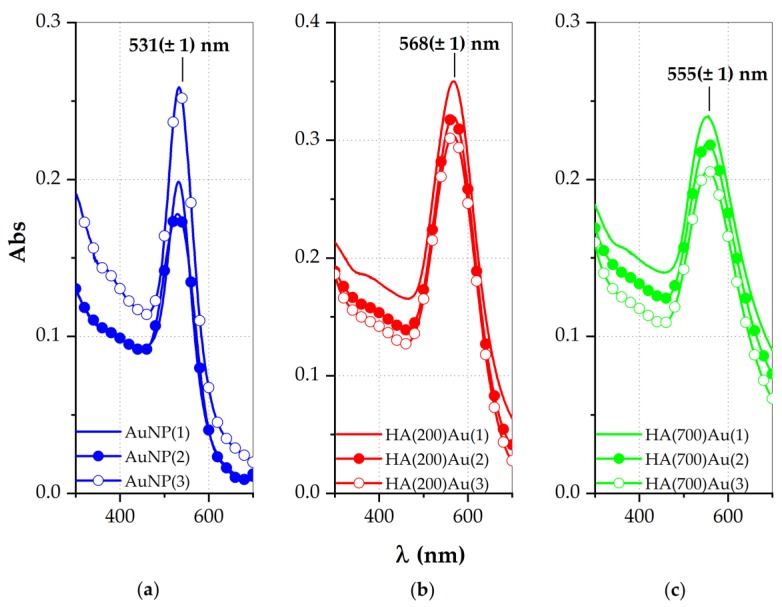
UV-vis spectra of gold nanoparticles (AuNPs): (**a**) as-prepared (‘fresh’) bare AuNPs, (**b**) HA200-conjugated AuNPs, (**c**) HA700-conjugated AuNPs. Each panel displays the spectra from three repeated syntheses.

In Figure 1 are displayed the spectra for three repeated syntheses of ‘bare’ (glucose-capped) AuNPs and hyaluronan-capped AuNPs, either 200 or 700 kDa, hereinafter named HA(200)Au or HA(700)Au, respectively.

The strong absorbance centered approximately at 531 nm for the AuNPs (Figure 1a) is red-shifted in the HA-AuNP hybrid samples. In particular, the plasmon peaks shift approximately at 568 nm for HA(200)Au (Figure 1b) and 555 nm for HA(700)Au (Figure 1c), respectively. Such shifts, expected upon the adsorption of molecules onto the nanoparticle surface [66], are explained by the interaction between the hyaluronan chains and the particles, which influence the surface plasmon resonance band of the gold nanoparticles [6].

The spectral parameters of the plasmon band, in terms of the full width at half maximum (FWHM), wavelength (λ_max_) and absorbance (A_max_) at the maximum for repeated syntheses (Table 1) highlight a good level of reproducibility and, in general, the formation of monodisperse colloidal suspension, as evidenced by FWHM values according to the literature data [67].

**Figure 2:**
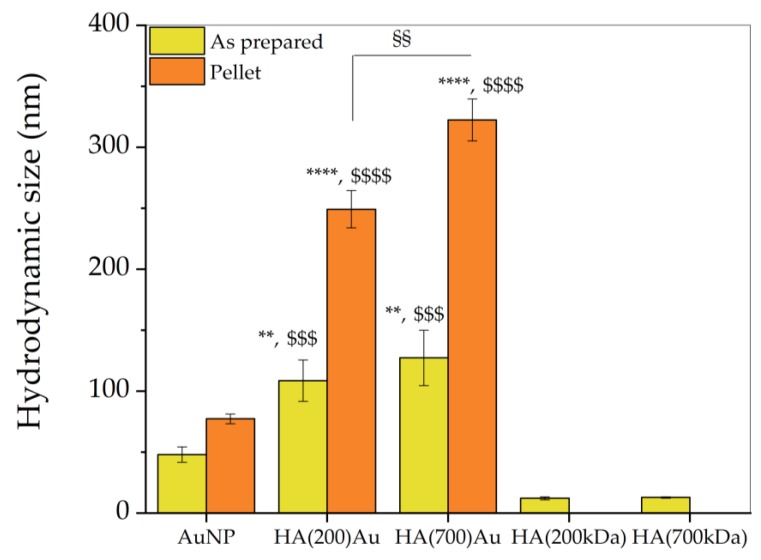
Hydrodynamic size of the bare AuNPs (as-prepared and ‘pellet 2’ samples) and HA-functionalized AuNPs (as-prepared and ‘pellet 3’ samples) measured by dynamic light scattering (DLS). (**) = *p* < 0.01, (****) *p* < 0.0001 vs. bare AuNP; ($$$) = *p* < 0.001, ($$$$) = *p* < 0.0001 vs. the control HA 200 or HA 700 (one-way ANOVA); (§§) = *p* < 0.01 vs. HA(200)Au.

Figure 2 shows the results of DLS analysis for the as-prepared and purified (see Materials and Methods) AuNP and HA-AuNP systems. The HA-coated NPs exhibit a larger size increase, likely due to a partial aggregation of the nanoparticles, than the bare AuNPs.

For the as-prepared nanoparticles, the hydrodynamic size of HA-capped AuNPs significantly increases compared to the bare, glucose-capped AuNPs, with a trend increasing with the MW of the hyaluronic acid.

Specifically, the DLS size of AuNPs (48 ± 6 nm) increased by roughly +125% for HA(200)Au (108 ± 17 nm) and +164% for HA(700)Au (127 ± 23 nm), respectively; no significant difference in the hydrodynamic size was found between the HA-capped NPs for the low- and high-MW hyaluronan polymers.

These findings can be figured out in terms of an actual size increase of the NPs, due to a shell made of hyaluronan chains wrapping the metallic Au core, and/or a partial aggregation effect. The latter is more likely to be predominant in the purified nanoparticles (pellet samples), where the hydrodynamic size increase compared to bare AuNP (77 ± 4 nm) is +223% for HA(200)Au (249 ± 15 nm) and + 318% for HA(700)Au (322 ± 17 nm).

Of note, for the pellets, the hydrodynamic size increase for HA(700)Au is significantly higher than the HA(200)Au samples, likely due to the formation of a more compact shell, and therefore a more efficient steric stabilization of the nanoparticle [6], formed by HA 200 kDa than the case of HA 700 kDa. It is indeed known that while oligomers adsorb to the nanoparticle surface and form a glassy layer in a molecular weight independent manner, the number of polymer chains with intermediate mobility between that of the glassy state and the bulk increases with molecular weight [68].

**Figure 3:**
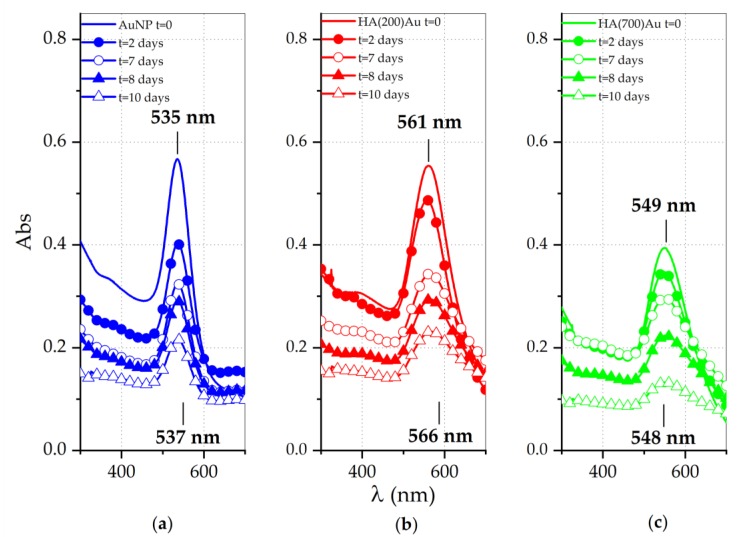
UV-vis spectra of AuNPs for the pellets collected after centrifugation and washing with ultrapure MilliQ water of: (**a**) as-prepared AuNPs (named ‘pellet 2’); (**b**,**c**) HA-conjugated NPs (named ‘pellets 3’; b: HA200, c: HA700). Each panel displays the spectra recorded at increasing aging times since the pellet resuspension up to 10 days.

To shed light on the aggregation effect due to the purification step, the plasmonic bands of the gold nanoparticles were monitored both for the freshly prepared pellets and during aging up to a time of 10 days (Figure 3 and Table 2).

The UV-vis spectra of freshly-prepared pellets of HA-capped AuNPs exhibit a blue shift in the plasmon wavelength at maximum absorbance compared to the spectra recorded for same samples before the purification, approximately of ~7 nm for HA(200)Au (Figure 3b vs. Figure 1b) and ~6 nm for HA(700)Au (Figure 3c vs. Figure 1c), respectively. On the contrary, the bare AuNPs pellets (Figure 3a) show a small red shift (~4 nm) in comparison to the as-prepared solutions (Figure 1a).

These findings can be explained in terms of either a digestive ripening effect by HA on the nanoparticle size evolution [69] prompted by stirring during the centrifugations for the purification of the NPs and/or by the removal of loosely bound polymer chains from the shell around the metallic core due to centrifugation and rinsing during the purification step.

Furthermore, results reported in Figure 3 and Table 2 also demonstrate a higher stability against aggregation during aging (associated with the spectral features both of red shifts as well as of increase in the FHHM of the plasmon peaks [70]) for bare AuNPs and HA(700)Au than for Ha(200)Au samples.

The experimental parameters displayed in Table 2 have been used to calculate the nanoparticle optical diameter (*d*, in nm) according to Equation (1) for AuNP [71]:

(1) $$d=\frac{\lambda \max-515.04}{0.3647}$$

whereas the molar extinction coefficient at the wavelength of maximum absorbance of the plasmon band (*ε_λ_*, in M^−1^·cm^−1^) was determined by using Equation (2) [72]:

(2) $$\varepsilon_{gold}=Ad^{\gamma }\;(d\;\leq \;85\;\mathrm{nm}:\;A=4.7\;\times \;10^{4};\;d\;>\;85\;\mathrm{nm}:\;A=1.6\;\times \;10^{8})$$

Taking into account the Lambert–Beer law, the molar concentrations of NP suspensions were calculated by assuming a spherical shape of the nanoparticle and a uniform face centered cubic (fcc) structure of the nanoclusters, with the length parameter of unit cell being L = 0.408 nm for AuNP, and the corresponding unit cell volume (*V_cell_* = L^3^) of 0.0679 nm^3^ [73]. By using Equation (3) to calculate the nanoparticle volume, $V_{NP}$:

(3) $$V_{NP}=\frac{4}{3}\;\pi \;{\left(\frac{d}{2}\right)}^{3}$$

and Equation (4) for the mole number of metal atoms for nanoparticle, $n_{NP}$:

(4) $$n_{NP}=4\cdot \frac{V}{V_{cell\cdot }NA}$$

where *N_A_* is Avogadro’s constant and 4 is the number of Au atoms that contains a one-unit cell, the nanoparticle concentration (in NP/mL) can be estimated by using Equation (5):

(5) $$NP/mL=\frac{c}{n_{NP}}$$

The optical diameter *d*, the molar extinction coefficient *ε* and the nanoparticle concentration values calculated by using the above-described equations for the pellets of Au nanoparticles are listed in Table 3 for both bare and HA-capped AuNPs at increasing aging times.

Table 3 shows that the calculated optical size matches quite well with the hydrodynamic diameter (d = 47 nm vs. 48 ± 6 nm) for as-prepared bare nanoparticles. On the other hand, for HA-capped AuNP samples, differently than the DLS results, the optical size for the HA(200)Au samples is higher than the value calculated for HA(700)Au.

According to the picture represented above, to explain the differences found in the hydrodynamic size depending on the hyaluronan MWs, and for the estimated optical size values, one can invoke the different chain ordering and arrangement by 200 or 700 kDa hyaluronic acid. Indeed, the optical diameter calculated from the plasmonic peak of the gold nanoparticles depends on the nanostructure morphology, the chemical composition, the electronic coupling strength, and, ultimately, on the fundamental coupling mechanisms [26]. On the other hand, the hydrodynamic size of a core-shell nanosystem is strongly affected by coordination and steric shielding capability of the macromolecules [74].

As for the change of the plasmonic peak features of the purified nanoparticles (pellet samples) with the aging, for both the bare and the high-MW HA-capped NPs there is no significant change detected, while for the low-MW HA-capped NP a significant increase in the optical size (~14 nm) is reached after one week.

**Figure 4:**
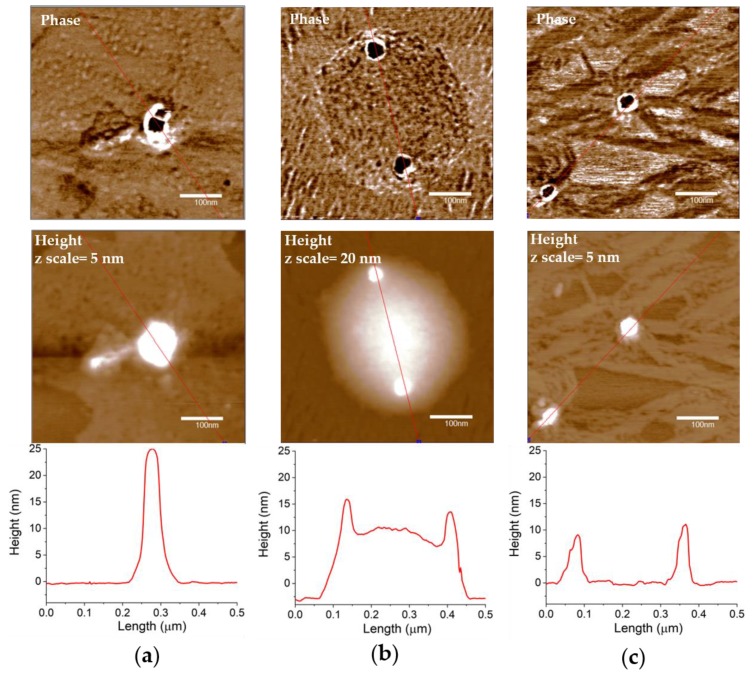
Atomic force microscopy (AFM) images recorded on a scan size of 500 × 500 nm^2^ in air AC mode of phase (upper panels) and height (middle panels) with related section analysis curves (lower panels) for: (**a**) bare AuNP, (**b**) HA-capped AuNPs with HA 200 kDa and (**c**) HA-capped AuNPs with HA 700 kDa.

Figure 4 displays the AFM micrographs, recorded in air AC mode, of bare and HA-coated gold nanoparticles. A metallic core (dark spherical features in the phase images) of similar size (~40 nm) is clearly visible for all three capping types, i.e., glucose, HA 200 kDa and HA 700 kDa. However, the structural arrangement of the polymer chains forming the shell around the metallic core highlights differences depending on the molecular weight of the hyaluronan, which in turn may affect the thickness and grafting density of the shell and consequently the stability of the nanoparticles.

In particular, the glycosidic matrix embedding the metallic nanoparticles (even if collapsed due to de-wetting driven effects upon the samples drying for imaging), is still compact around the metallic core for glucose-capped AuNPs (Figure 4a) and HA(200)Au samples (Figure 4b), while uncoiled ribbons of polymer are detected for HA(700)Au (Figure 4c).

**Figure 5:**
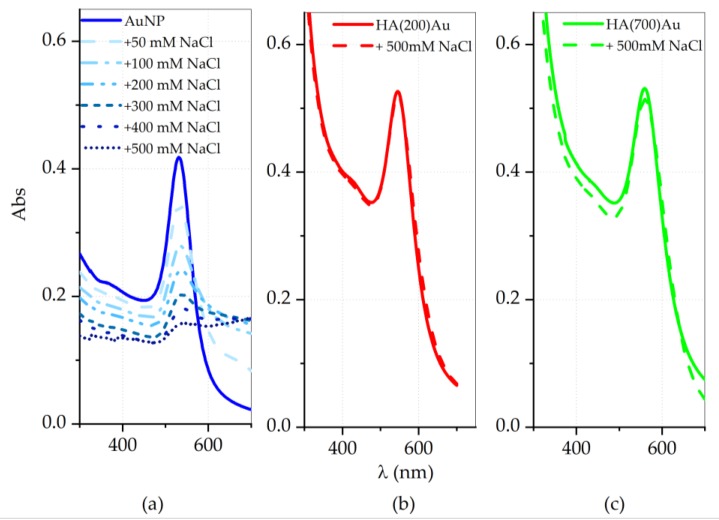
UV-visible spectra of bare AuNP (**a**) before (solid line) and after (dashed lines) the gradual addition of NaCl aqueous solution at increasing concentrations from 0.05 M to 0.5 M. The UV-visible spectra before (solid line) and after (dashed lines) the direct addition of 0.5 M NaCl are shown in (**b**) for HA(200)Au (0.11 nM = 4 × 10^3^ AuNP/mL) and in (**c**) for HA(700)Au (0.06 nM = 6 × 10^2^ AuNP/mL).

**Figure 6:**
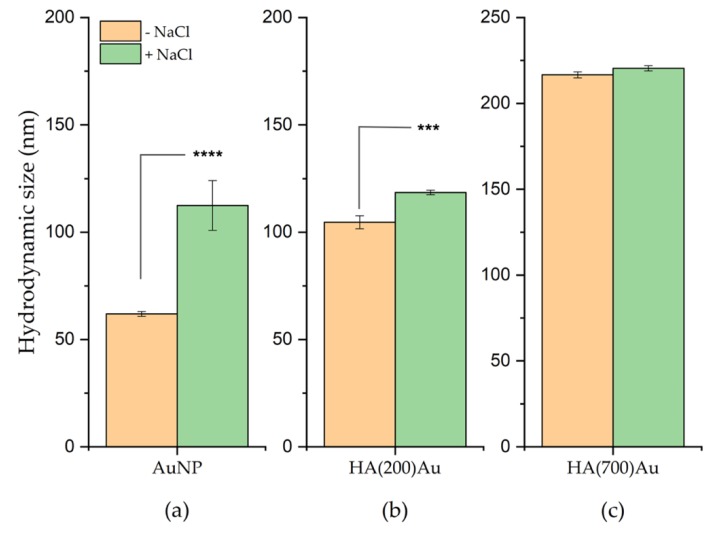
DLS measurements of the hydrodynamic size of nanoparticles before and after the addition of 0.6 M NaCl to: (**a**) bare AuNP, (**b**) HA (200)Au, (**c**) HA(700)Au. (***) = *p* < 0.001 and (****) = *p* < 0.0001, vs. the corresponding sample not added with the electrolyte solution (one-way ANOVA).

The effect of the hyaluronan coating on the nanoparticle stability against aggregation due to electrostatics was investigated through flocculation assay, by addition of a strong electrolyte (NaCl) to the nanoparticle suspensions, and by monitoring, both before and after the addition, the changes in the plasmonic peaks by UV-visible spectroscopy (Figure 5) and in the hydrodynamic size by DLS (Figure 6), respectively.

For both HA(200)Au and HA(700)Au, the capping of the gold nanoparticle core with hyaluronan is efficient to stabilize them against aggregation due to the disruption of electrostatic mechanism of stabilization [6].

The bacterial assays with the treatments of Gram-negative *E. coli* ATCC9637 and Gram-positive *S. aureus* ATCC 29213 by AuNP, HA(200)Au and HA(700)Au samples demonstrated comparable results of inhibitory activity against the two bacterial strains, as tested by using the broth microdilution method. Table 4 shows the highest inhibitory activity for HA(700)Au (MIC (minimum inhibitory concentration) value of 1.0 × 10^−5^ µg/mL, corresponding to 0.4 nM concentration of gold nanoparticles = 1.1 × 10^7^ NP/mL), followed by HA(200)Au (MIC value of 1.4 × 10^−5^ µg/mL, corresponding to 0.6 nM concentration of gold nanoparticles = 5.9 × 10^6^ NP/mL) and, ultimately, by AuNP (MIC value of 1.4 × 10^−1^ µg/mL corresponding to 0.6 nM concentration of gold nanoparticles = 7.0 × 10^7^ NP/mL).

**Figure 7:**
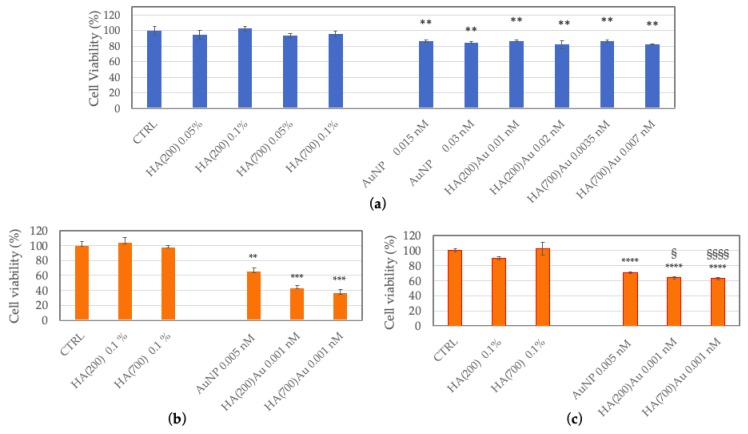
Viability of HUVEC (**a**), SH-SY5Y (**b**) or PC-3 (**c**) cells, untreated (CTRL) and after 24 h of treatment with bare AuNP, HA(200)Au, HA(700)Au or the positive controls of HA 200kDa or HA 700 kDa. (**) = *p* < 0.01, (***) = *p* < 0.001, (****) = *p* < 0.0001 vs. CTRL; (§) = *p* < 0.05, (§§§§) = *p* < 0.0001 vs. AuNP (one-way ANOVA). Average values ± S.E.M. from experiments repeated in triplicate.

The cytotoxicity response of both normal and tumor cells was investigated on three human model cell lines, namely endothelial (HUVEC), neuroblastoma (SH-SY5Y) and prostate (PC-3) cells. Figure 7 shows the cell viability, determined by MTT assay, for the cells incubated for 24 h with AuNP, HA(200)Au or HA(700)Au samples. The negative control of untreated cells and the positive controls of cells treated with HA 200 kDa or HA 700 kDa are shown for comparison.

In the CD44-positive normal endothelial cells (Figure 7a), for all treatments, only a negligible toxicity (~20% decrease in cell viability with respect to untreated cells) was detected, with no dose-dependent effects found. A slightly higher toxicity was instead observed for CD44-positive prostate tumor cells (Figure 7c), with a decrease in cell viability compared to untreated cells of ~ 30% for cells treated with bare AuNPs and ~ 35% for treatments with HA-capped NPs, respectively. Finally, the highest toxicity was detected in CD44-negative neuroblastoma cells (Figure 7b), with a decrease of viability of ~40% and ~60% for treatments with bare AuNP and HA-capped NPs, respectively.

In order to shed light on the receptor-dependent and MW-dependent cellular uptake of the hybrid HA–AuNP systems, we performed confocal microscopy imaging on the CD44-negative neuroblastoma and the CD44-positive prostate cancer cells upon treatments (90 min of incubation time) with bare and HA-capped AuNPs.

**Figure 8:**
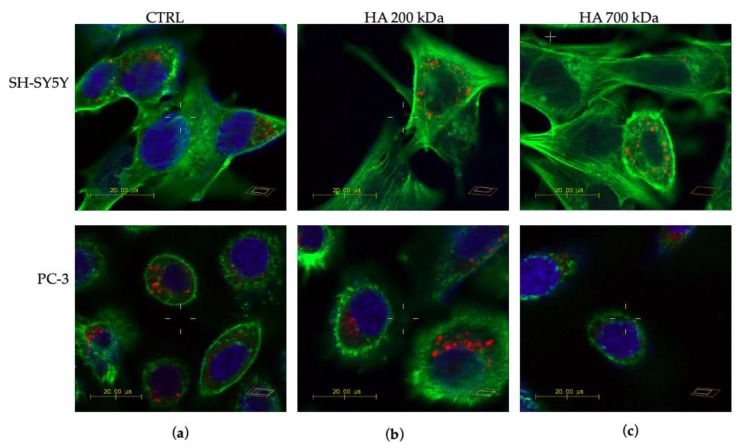
Laser scanning microscopy (LSM) representative micrographs of neuroblastoma (SH-SY5Y) and prostate (PC-3) tumor cells, untreated (**a**) or treated by 90 min incubation with HA 200 kDa (0.1 % *w*/*v*) (**b**) or HA 700 kDa (0.1 % *w*/*v*) (**c**). Each panel displays the merged confocal images of nuclear (in blue, Hoechst, λ_ex/em_ = 405/425–450 nm), cytoskeleton actin (in green, Actin Green, λ_ex/em_ = 488/500–530 nm) and lysosomal (in red, Lysotracker Red, λ_ex/em_ = 543/550–600 nm) staining. Scale bar = 20 µm.

**Figure 9:**
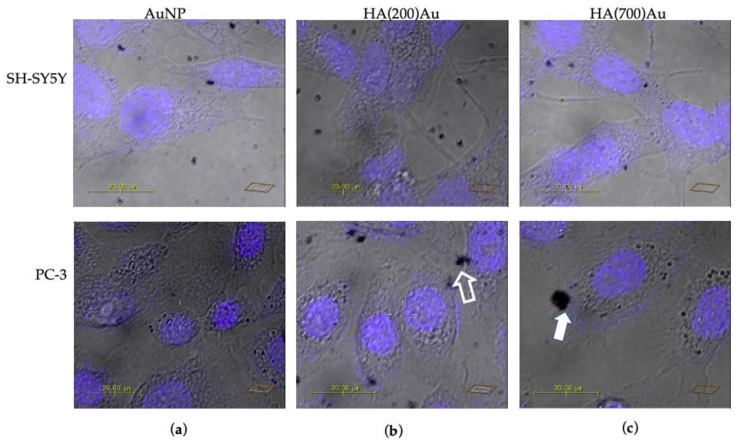
Merged confocal images of Hoechst staining (λ_ex/em_ = 405/425–450 nm) + optical bright field micrographs of neuroblastoma (SH-SY5Y, upper panels) and prostate cancer (PC-3, lower panels) cells dye-labelled and fixed after 90 min of incubation with: (**a**) AuNP (0.005 nM = 5.5 × 10^2^ NP/mL); (**b**) HA(200)Au (0.0010 nM= 7.2 NP/mL, 0.1% *w*/*v* HA); (**c**) HA(700)Au (0.0011 nM = 2.5 × 10^1^ NP/mL, 0.1% *w*/*v* HA). Scale bar = 20 µm. The arrows point to gold nanoparticle aggregates (dark features in the optical micrographs).

**Figure 10:**
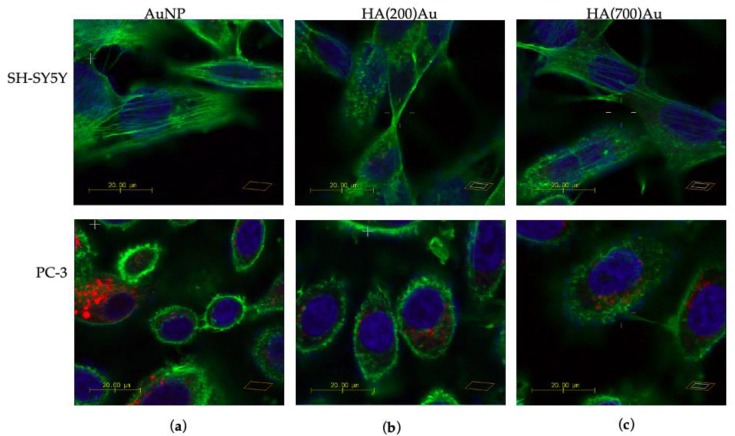
LSM merged confocal micrographs of neuroblastoma (SH-SY5Y, upper panels) and prostate cancer (PC-3, lower panels) cells: in blue, the nuclear staining with Hoechst (λ_ex/em_ = 405/425–450 nm); in green, the cytoskeleton actin staining with Actin Green (λ_ex/em_ = 488/500–530 nm); in red, the lysosomal staining with Lysotracker Red (λ_ex/em_ = 543/550–600 nm). Cellular treatments were carried out for 90 min with (**a**) AuNP (0.005 nM = 5.5 × 10^2^ NP/mL); (**b**) HA(200)Au (0.0010 nM= 7.2 NP/mL, 0.1% *w*/*v* HA); (**c**) HA(700)Au (0.0011 nM = 2.5 × 10^1^ NP/mL, 0.1% *w*/*v* HA). Scale bar = 20 µm.

Figure 8, Figure 9 and Figure 10 show the representative confocal microscopy (in blue, the nuclear staining with Hoechst; in green, the cytoskeleton actin staining with Actin Green; in red, the lysosomal staining with Lysotracker Red, respectively) and the optical bright field (in grey) micrographs for the two different cellular models.

A cell-specific response can be observed in the LSM micrographs after treatment with hyaluronic acid in comparison to the negative control (Figure 8a). In particular, in the neuroblastoma SH-SY5Y cells (Figure 8b,c, upper panels), the Hoechst staining of the nuclei appeared dim, which suggests a diffuse and massive uptake of the hyaluronan in the whole cytoplasm, for both the treatments with HA 200 kDa or HA 700 kDa. It is, indeed, very likely that, upon the cellular internalization of hyaluronic acid, the viscoelastic properties of the hydrogel can induce a ‘wrapping’ effect of the intracellular organelles, thus making less effective the dye staining. Such ability of HA to enter the cell nuclei was also demonstrated on fibroblast cells in which the presence of HA in the nucleus was associated with the appearance of cleft and furrows as well as nucleoli [75]. On the other hand, prostate tumor PC-3 cells after incubation with hyaluronic acid still show the characteristic compact shape with near-circular geometry and the nucleus located at approximately the center of the cell (Figure 8b,c, lower panels). However, while the cytoskeletal actin in the control untreated cells is observed only at the cell periphery, for the cells incubated with HA 200 kDa or HA 700 kDa an extended amorphous actin meshwork is well defined throughout the whole cell body.

Figure 9 clearly shows the uptake of the gold nanoparticles by the two cellular models used, as displayed by the dark features in the optical bright field micrographs, which correspond to nanoparticle aggregates. Of note, for the CD44-negative neuroblastoma (Figure 9, upper panels), the aggregate distribution is quite similar in the cells incubated with bare AuNPs (Figure 9a) or with HA-capped NPs (Figure 9b,c). Furthermore, no significant changes in lysosomal staining or in the cytoskeleton actin were found. On the other hand, in the CD44-positive prostate cancer cells (Figure 9, lower panels), large nanoparticle aggregates are accumulating at the cell membrane of cells treated with HA(200)Au samples (see, for instance, the open arrow in Figure 9b) and even larger aggregates are visible for those treated with HA(700)Au samples (Figure 9c, solid arrow).

**Figure 11:**
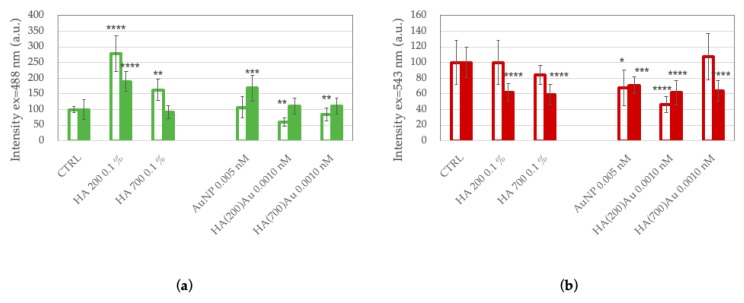
Analysis of emission intensity for cytoskeleton actin (**a**) and lysosomal (**b**) staining of CD44-negative SH-SY5Y (open bars) and CD44-positive PC-3 (solid bars) tumor cells. Cellular treatments were carried out for 90 min with (**a**) AuNP (0.005 nM = 5.5 × 10^2^ NP/mL); (**b**) HA(200)Au (0.0010 nM= 7.2 NP/mL, 0.1% *w*/*v* HA); (**c**) HA(700)Au (0.0011 nM = 2.5 × 10^1^ NP/mL, 0.1% *w*/*v* HA). (*) = *p* < 0.05, (**) = *p* < 0.01, (***) = *p* < 0.001, (****) = *p* < 0.0001 vs CTRL (one-way ANOVA). Average values ± S.E.M. from randomly chosen areas of micrographs from experiments repeated in triplicate.

The confocal micrographs of cells dye-labelled at the nuclei (in blue), the cytoskeleton actin (in green) and the lysosomes (in red) also show different responses in the intracellular organelle staining after the treatments with bare AuNP and the HA-capped AuNP samples for SH-SY5Y and PC-3 cells, respectively (Figure 10 and Figure 11).

In particular, for CD44-negative SH-SY5Y cells (Figure 10, upper panels) a rearrangement of actin stress-fibers is visible, with globular features (actin dots) that become predominant after treatment with HA(200)Au (Figure 10b) or HA(700)Au (Figure 10c) with respect to filamentous F-actin structures observed in the cells incubated with bare AuNPs (Figure 10a). The quantitative analysis of fluorescence emission (Figure 11a) shows a decrease in the actin fiber intensity for SH-SY5Y cells treated with HA-capped NPs compared to the untreated cells, which suggests an actin depolimerization of actin fibers prompted by the hybrid HA-AuNP samples. It is noteworthy that the opposite trend is exhibited by the SH-SY5Y cells treated with low- or high-MW HA, with an increase in the actin fiber intensity compared to the untreated cells.

As to the CD44-positive PC-3 cells (Figure 10, lower panels), a similar assembly of cytoskeletal actin filaments along the cell periphery is found for the cells incubated with bare AuNP or HA(200)Au. To note, such an assembly of actin structures is thicker than that observed for the control untreated cells (see Figure 8a). On the contrary, for the prostate cancer cells treated with HA(700)Au samples, a dense actin network is found, similar to that promoted by the cell incubation with HA 700 kDa alone (see Figure 8c). The quantitative analysis of the cytoskeleton actin in PC-3 cells does not show significant changes in the F-actin fiber intensity except in the treatments with HA 200 kDa and AuNPs.

For both SH-SY5H and PC-3 cell lines, no significant changes after the different treatments could be appreciated in the morphological features of the lysosomal pattern (Figure 10). The quantitative analysis (Figure 11b) shows, especially for the PC-3 cells, a general decrease in the emission intensity of the lysosomal tracker for all the conditions of treatment compared to the untreated cells.

## 3. Discussion

Several research studies have focused on developing new green-like strategies to obtain gold nanoparticles at mild temperature conditions. For example, Jung et al. synthesized at room temperature gold nanoparticles from auric acid with glycosides as reducing agents in aqueous NaOH [76]. More recently, Suvarna et al. synthesized AuNPs differently capped with glucose, 2-deoxy-d-glucose and citrate, used both as reductants and as capping agents by a one-step room temperature procedure. In the same work the authors tested three types of AuNPs in three different cancer cell lines and did not find any toxicity [77]. In another report, carbohydrate-decorated gold nanoparticles were prepared by using phosphino aminoacid P(CH_2_NHCH(CH_3_-)COOH)_3_ as a reducing agent and a reaction temperature of 80 °C to coat the metal core of the nanoparticle with a monosaccharide (glucose), disaccharides (sucrose, maltose, or lactose), a trisaccharide (raffinose) and a polysaccharide (starch) [78].

The advantage of this approach is to get core-shell structures of bioconjugated nanoparticles with enhanced biocompatibility and, in some cases (such as the glucose-capped AuNPs) a targeting capability towards cancer cells. To this respect, hyaluronan HA-capped gold nanoparticles have been extensively exploited for active tumor targeting, due to the specific interaction of this polymer with the CD44 receptor, which is overexpressed in several cell lines [44].

**Figure 12:**
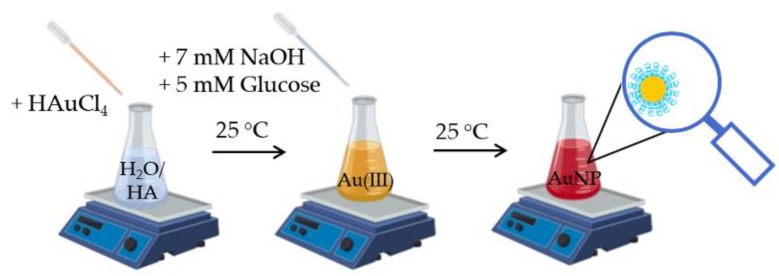
Schematic illustration of the synthesis of HA-capped AuNPs.

In the present study we addressed a novel synthesis of HA-capped AuNPs with a green, reproducible one-step process (Figure 12) and scrutinized the nanoparticle physicochemical features, such as hydrodynamic and optical size, as well as the aging stability, related to the different molecular weights of the used hyaluronan polymers, namely 200 and 700 kDa.

For the HA-capped AuNPs we detected a red shift in the plasmonic band compared to the bare, glucose-capped AuNPs (Figure 1), explained as due to both the increase in the optical size of the nanoparticles and/or a partial aggregation effect (Table 1) [6,79]. According to the literature [80], we found that the decoration of the gold nanoparticles by low- or high-MW HA resulted in correlated different hydrodynamic sizes of the HA–AuNP hybrids (Figure 2), respectively.

Of note, the hydrodynamic size includes both the size of the core gold nanoparticle and the thickness of the swelled polymer shell around it; a simple model to predict the trends in nanoparticle diameter increase is that the steric bulk of the polymeric chains, obviously related to the their length and thus the molecular weight of HA, protect the nanoparticles from aggregation [6].

We actually found a comparably high stability against aggregation induced by the disruption of the electrostatic mechanism of colloidal stabilization for both HA(200)Au and HA(700)Au samples, as investigated by UV-visible (Figure 5) and DLS (Figure 6) analyses, respectively. On the other hand, the spectroscopic characterization of the recovered pellets of the purified nanoparticles, for both the freshly prepared samples and those aged up to 10 days (Figure 3, Table 3), pointed to different aging stabilities for the low- and the high-MW HA-capped NPs. In particular, the calculated optical size changes pointed to a more compact structural arrangement of the polymer chains around the metallic core for HA(200)Au than HA(700)Au, which in turn may affect the thickness and grafting density of the shell. This model was confirmed by the analysis of phase and height AFM micrographs recorded in air AC mode (Figure 4), unraveling a still intact glycosidic matrix embedding the metallic nanoparticles of bare (glucose-capped) and low-MW HA-capped NPs but polymer ribbons uncoiled from the metallic core for the high-MW HA-capped AuNPs.

The use of hyaluronan polymers at different MW allowed for scrutinizing the potential of these hybrid systems as tunable anti-angiogenic theranostic platform. Indeed HA, a major glycosaminoglycan of the extracellular matrix, has cell-signaling functions that depend on its molecular weight: anti-inflammatory and anti-angiogenic effects for high-MW HA and pro-inflammatory effects for low-MW HA, respectively [81].

To note, the binding affinity of HA to CD44 receptors is known to be highly dependent on the molecular weight of the HA; higher MW HA has a higher binding affinity than lower MW HA [82]. Furthermore, Wolny et al. found that the binding between HA with a MW higher than 262 kDa and CD44 is strong and irreversible, while the binding between HA with a MW lower than 262 kDa is much weaker and reversible [83].

Because of their comparability in size to the distance between cell-surface targets, AuNPs can simultaneously engage multiple adjacent receptor sites, thus achieving increased uptake in the cells. Hence, as the hydrodynamic dimension of AuNPs increases after the functionalization with hyaluronic acid, an increased binding specificity to CD44 receptor is expected, as well as an enhancement of their cytotoxic effects due to the improved targeting capability and the increased cellular uptake depending on the HA coating.

To support and validate our model of increased cytotoxicity for HA-coated AuNPs, we performed antibacterial assays on Gram-negative *E. coli* and Gram-positive *S. aureus*. Our HA-capped AuNPs exhibited an enhanced inhibitory activity against *Escherichia coli* ATCC 9637 and *Staphylococcus aureus* ATCC 29213 as well as a better targeted cytotoxicity towards CD44-positive tumor cells than the bare, glucose-capped, AuNPs (Table 4).

The cytotoxicity of AuNPs against cancerous cells occurs owing to the highest uptake of nanoparticles by these cells rather than healthy cells, given that cancerous cells have an abnormal metabolism and high proliferation rate, which in turn makes them more vulnerable [23]. However, gold nanoparticles do not universally target any cell type, which may explain the need to target specific tumor lines, as in the case of hyaluronan-coated nanoparticles that target the cell membrane overexpressing the CD44 receptor [84]. The surface functionalization of NPs with HA, due to the presence of –COOH functional groups, increases the negative charge of NPs and, according to the literature data, enhance their uptake [85]. Indeed, polysaccharide-conjugated NPs tend to be individually distributed and thus have freedom of access within the cell and a larger surface area available for interaction with cellular constituents [86].

We tested the viability after 24 h of treatment with bare or HA-capped AuNPs of CD44-positive cells (normal endothelial, HUVEC and prostate tumor, PC-3), in comparison with CD44-negative cells (neuroblastoma, SH-SY5Y) (Figure 7).

Flow cytometry and Western blot experiments demonstrated high levels of CD44v6 expression in PC-3 cells [87] and that CD44 is not expressed or is expressed at greatly reduced levels in human neuroblastoma lines [88]. Moreover, CD44 silencing experiments in prostate cancer were performed to demonstrate that the loss of this receptor activity is associated with tumor recurrence and progression [89]; also, the majority of prostate cancer metastases lack the expression of this molecule [90].

Only a negligible toxicity (~20% decrease in cell viability with respect to untreated cells) was detected in HUVEC, with no dose-dependent effects for all treatment conditions. A slightly higher toxicity was instead observed for PC-3 (~35% and ~30% decrease in cell viability compared to untreated cells for treatments with bare and HA-capped NPs, respectively). Finally, the highest toxicity was detected in neuroblastoma cells, with a decrease of viability of ~40% and ~60% for treatments with bare AuNP and HA-capped NPs, respectively.

**Figure 13:**
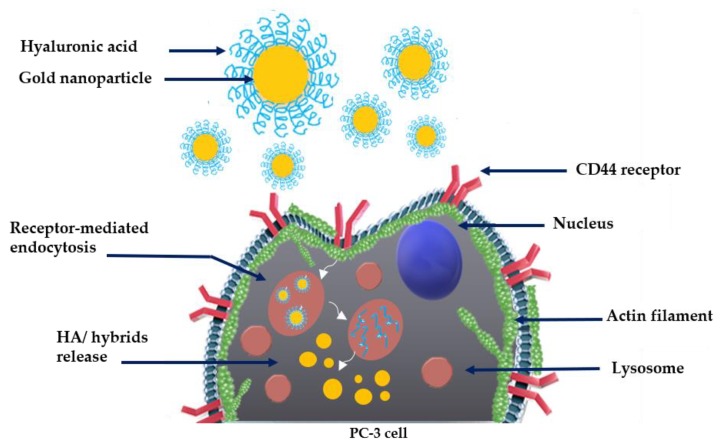
Schematic illustration of cellular uptake and intracellular trafficking: HA–AuNP hybrids interaction with lysosomes and cytoskeletal actin via CD44–HA site-specific binding on PC-3 tumor cell.

The link between hyaluronic acid and the CD44 adhesion molecule can initiate a series of events that begin with matrix adherence modifications and continue with the activation of other molecules (such as growth factors), degradation of the matrix itself, angiogenesis, vessel permeation and extravasation [49]. All these steps are necessary in the initiation of the metastatic pathway [50,91]. It has been demonstrated that, from an oncological point of view, CD44 has a considerable importance in the study of progression and tumor invasiveness. An invasive tumor in fact, to expand, attacks the extracellular matrix of the surrounding tissues and CD44, together with hyaluronic acid, certainly plays a decisive role in the various cellular pathways (Figure 13) [92].

Confocal microscopy imaging on the CD44-negative neuroblastoma and the CD44-positive prostate cancer cells upon treatment (90 min of incubation time) with bare and HA-capped AuNPs highlighted some receptor-dependent and MW-dependent features in the cellular uptake of the hybrid HA-capped systems compared to the positive controls of bare NPs and HA (both low- and high-MW) (Figure 8, Figure 9, Figure 10 and Figure 11).

At the leading edge of a motile cell, actin polymerizes in close apposition to the plasma membrane. Actin assembles at the membrane into a dense network, together with proteins that regulate the branching, cross-linkage and membrane anchorage of the actin filaments [78].

Our results on CD44-positive PC-3 and CD44-negative SH-SY5Y cells showed different patterns of F-actin organization. Specifically, for PC-3, we found: (i) an assembly of stress fiber actin confined at the cell periphery in untreated cells and thickened in the cells incubated with bare AuNP or HA(200)Au; (ii) an extended actin meshwork, for cells incubated with HA 200 kDa, HA 700 kDa or HA(700)Au samples. On the other hand, for SH-SY5Y cells, the treatment with HA-capped AuNPs promoted the F-actin depolimerization with the formation of actin dots.

According to the literature, actin F-fibers can be disrupted to various extents depending on the aggregation state of Au NPs, with varying decreases in F-actin fiber intensity and thickness and appearance of actin dots [79]. On the other hand, high-MW-HA may promote lamellipodia formation, actin stress-fiber arrangement and cell migration (but not proliferation) [77]. To note, upon the activation of the CD44 receptor, PC-3 cells are known to increase their migration and invasion showing adhesive morphology with extensive cell spreading and invadopodia formation as well as organized actin-stress fibers [80].

As for lysosomes, for both SH-SY5H and PC-3 cell lines, we did not detect significant changes after the different treatments in the morphological features of the lysosomal pattern. However, especially for PC-3 cells, a general decrease in the emission intensity of the lysosomal tracker was found for all conditions of treatment compared to the untreated cells. It is known that AuNPs aggregate in autophagic vesicles due to their biological stability, which leads to lysosomal swelling and impaired autophagy flux through shape-dependent endocytosis and subsequent lysosomal dysfunction [81]. Experiments on CD44-positive human fibroblasts cells showed a very quick (about 60% of internalized HA after 20 min of incubation) cellular uptake of HA and its accumulation into lysosomes, with a partially CD44-independent mechanism [93].

## 4. Materials and Methods

### 4.1. Chemicals

Sodium hyaluronate (HA-200 kDa and 700 kDa) was a kind gift from Fidia Farmaceutici S.p.A (IT). d-(+)-glucose purity ≥ 99.5%, gold (III) chloride trihydrate (HAuCl_4_) purity ≥ 99.9 % and sodium hydroxide (NaOH), were purchased from Sigma-Aldrich. 3-(4,5-dimethylthiazol-2-yl)-2,5-diphenyltetrazolium bromide (MTT reagent), Dulbecco’s modified eagle medium (DMEM F-12) high glucose, RPMI-1640 and fetal bovine serum (FBS) were purchased from Sigma-Aldrich (St. Louis, MO). Ultrapure Milli-Q water was used (18.2 mΩ·cm at 25 °C, Millipore).

### 4.2. Synthesis of Bare and HA-Conjugated AuNP

Bare metal NPs were synthesized by using ultrapure water as solvent, according to Equation (6):

(6) $$3C_{6}H_{12}O_{6\;}+6OH^{-}+2Au^{3+}\to 3C_{6}H_{12}O_{7\;}+2Au^{0}+H_{2}O$$

As to the synthesis of bare AuNP in MilliQ-H_2_O, the following aqueous stock solutions were prepared at room temperature: 1.0 mM chloroauric acid (prepared by adding 5 μL of 1 M of HAuCl_4_ to 995 μL of H_2_O-MilliQ), 100 mM sodium hydroxide (160.0 mg, corresponding to 4.0 mmol of NaOH dissolved in 40 mL MilliQ-H_2_O) and 100 mM glucose (0.180 g of C_6_H_12_O_6_ dissolved in 10 mL of MilliQ-H_2_O). Briefly, 0.1 mL of 1 mM HAuCl_4_ (final concentration 0.5 mM) was diluted in 0.79 mL of MilliQ-H_2_O, added with 0.07 mL of 100 mM NaOH (final concentration 7 mM) for basic condition and 0.05 mL of 100 mM glucose (final concentration 5 mM) as a reducing agent. The synthesis produces a particle with a plasmonic peak centered at 540–560 nm, according to the literature [53]. As to the synthesis of HA-conjugated NPs, firstly the hyaluronic acid was dissolved in MilliQ-H_2_O: 10 mg of HA200 or HA700 was weighed and solubilized in 2.5 mL of MilliQ-H_2_O, under constant magnetic stirring for 2 h, to obtain the 0.4% (*w*/*v*) stock solution. For the synthesis of HA-conjugated AuNP, the HA200 (or HA700) stock solution 0.4 % (*w*/*v*) (final concentration in the mixture reaction of 0.2 *w*/*v*), was placed in Eppendorf ThermoMixer at controlled temperature of 25 °C. Briefly, 0.1 mL of HAuCl_4_ 1 mM (final concentration = 0.5 mM) was added to 0.79 mL of HA 200 (or HA 700). Finally, to obtained solutions were added 0.07 mL of 100 mM NaOH (final concentration = 7 mM) for basic condition and 0.05 mL of 100 mM glucose (final concentration = 5 mM) as a reducing agent. The AuNPs were formed immediately [76]. The as-prepared AuNP solutions in water were washed by two centrifugation steps in an Eppendorf Centrifuge 5424 for 15 min at 6800 RCF, with one rinsing with H_2_O-MilliQ in between the centrifugations, to remove excess unreacted reducing agent as well as to concentrate the nanoparticle dispersion (‘pellet 2’). The NP-HA samples were centrifuged twice through with Amicon Ultra-0.5 mL Centrifugal Filter devices 30 K (Merk, Millipore, Burlington, MA, USA), at 9391 RCF for about 4 min, with two washings with MilliQ-H_2_O in between the centrifugation steps, to remove the excess reducing agent from the nanoparticles dispersion and to concentrate the nanosystems ‘pellet 3’.

### 4.3. UV–Visible (UV-vis) Spectroscopy and Dynamic Light Scattering (DLS) Analysis

UV-vis spectroscopy was performed on the aqueous dispersions of AuNPs, in quartz cuvettes with 0.5 and 0.1 cm optical path length on a Perkin Elmer UV-vis spectrometer (Lambda 2S, Waltham, MA, USA). The dynamic light scattering (Horiba LB-550, Kyoto, Japan) was used to characterize the surface charge and the size distribution of the nanoparticles and the results were presented as the mean of at least three measurements

### 4.4. Bacterial Assays

#### 4.4.1. Strains and Culture Conditions

The bacteria tested in this study were the reference strains *Escherichia coli* ATCC 9637 and *Staphylococcus aureus* ATCC 29213, belonging to the collection of Bacteriological Laboratory of Fidia Farmaceutici S.p.A. (Noto, Italy). Both strains were grown in Mueller Hinton (MH, Oxoid, Basingstoke, Hants, UK) broth and agar and incubated at 37 °C under aerobic conditions (Memmert incubator, GmbH + Co. KG, Äußere Rittersbacher Straße) for 18–24 h.

#### 4.4.2. Broth Microdilution Assay

To determine the inhibitory capability of NPs the broth microdilution assay was performed in agreement with CLSI M27-A3 and CLSI M100-S23 [94]. Briefly, the NPs were dispensed in 96-well plates (Sigma-Aldrich, St. Louis, MO, USA) and diluted in MH broth performing serial two-fold dilutions, as follows: AuNPs from 0.585 nM (= 1.45 × 10^−1^ µg/mL) to 0.07 (= 1.81 × 10^−6^ µg/mL); HA200-AuNPs from 0.55 nM (= 1.37 × 10^−5^ µg/mL) to 0.07 nM (= 1.71 × 10^−6^ µg/mL); HA700-AuNP from 0.417 nM (= 1.03 × 10^−5^ µg/mL) to 0.05 nM (= 1.29 × 10^−6^ µg/mL); chloramphenicol was tested at the concentration range from 124 to 0.5 µg/mL. For each strain the inoculum was prepared suspending individual colony, pre-cultured overnight (16–18h) on MH agar plates in 5.0 mL of sterile saline solution (NaCl 0.95% *w*/*v*). The density of the suspension was adjusted to achieve a turbidity of 0.5 McFarland (1.0–2.0 × 10^8^ CFU/mL, OD_630_ of 0.16–0.2, measured using Bioteck Synergy HT spectrophotometer). The bacterial suspension was then diluted to obtain in each well of the 96-well plate a final concentration of about 5.0 × 10^5^ CFU/mL, after inoculum. MH broth (Oxoid, Basingstoke, Hants, UK) inoculated with the tested strain was used as a positive control and sterile MH broth was used as negative control. The 96-well plates were incubated at 37 °C for 24 h in aerobic conditions, according to CLSI M100-S23. In order to determine the inhibitory activity of tested NPs, the minimum inhibitory concentration (MIC) was determined following the guideline of CLSI M7-A7. The MIC was the lowest concentration of NPs that completely inhibited visible bacterial growth in the well, compared with the positive control. The bacterial growth inhibition was confirmed by spreading on MH agar (Oxoid, Basingstoke, Hants, UK) 100 µL from each well in which the bacterial growth appeared inhibited. The experiments were carried out two times in duplicate. The results are expressed reporting the NPs concentration as nanomolar (nM) and µg/mL which is the standard unit for the MIC measurements, to facilitate the comparison with other data reported in the literature.

### 4.5. Cell Cultures and Maintenance

Human neuroblastoma SH-SY5Y and prostate cancer (PC-3) cell lines were cultured in 25 cm^2^ plastic flasks in DMEM F-12 and RPMI-1640, respectively. Both mediums were supplemented with 10% *v*/*v* foetal bovine serum (FBS) and contained 2 mM l-glutamine, 50 IU/mL penicillin and 50 µg/mL streptomycin. Cells were grown in tissue-culture-treated Corning^®^ flasks (Sigma-Aldrich, St. Louis, MO, USA) in an incubator (Heraeus Hera Cell 150C incubator), under a humidified atmosphere at 37 °C in 5% CO_2_. For the cellular treatments, the day before the experiment, cells were seeded in full medium on TPP^®^ tissue culture plates (Sigma-Aldrich, St. Louis, MO, USA). The HA-conjugated gold nanoparticle samples were concentrated 5 times up to the final HA concentration of 1% *w*/*v* (‘pellet 3’) or, in the case of the bare AuNP reference samples, up to the maximum washing by centrifugation steps to avoid aggregation (typically ‘pellet 2’ samples), and added to the cells in culture medium at the desired final concentration for incubation times of 90 min (confocal microscopy experiments) or 24 h (cell viability assays), respectively.

#### 4.5.1. Cell Viability Assay

For MTT assay, cells were seeded at a density of 12,000 cells/well and 10,000 cells/well, respectively, for SH-SY5Y and PC-3 and maintained in their respective complete media in standard culture condition. The day after, cells were washed with 1 % FBS-supplemented medium and treated with samples (HA-conjugated NPs and bare NPs) with a 10X dilution of pellet samples. After 24 h of incubation, cells were washed with buffer and treated with 5 mg/mL of 3-(4,5-Dimethyl-2-thiazolyl)-2,5-diphenyl-2H-tetrazolium Bromide (Sigma-Aldrich (St. Louis, MO, USA) at 37 °C for 90 min. At this stage, the formazan salts formed by succinate dehydrogenase activity in live cells were solubilized with DMSO and quantified spectrophotometrically by a Synergy 2 microplate readers (BioTek, Winooski, VT, USA), by the absorbance value at 570 nm of wavelength. All conditions were measured in triplicate and results were expressed as % of viable cells over the negative control (i.e., untreated cells).

#### 4.5.2. Confocal Microscopy Analysis

For LSM live cell imaging analysis, cells were seeded at a density of 25.000 cells/well in 12 mm glass bottom dishes (Willco Wells, Amsterdam, The Netherland). The day after cells were washed with 1% FBS-supplemented medium and treated with HA-conjugated NP and bare NP samples for 90 min. During the last 20 min of the total treatment period, the cells were stained with LysoTracker Red (150 nM), a red-fluorescent dye for labeling and tracking acidic lysosome organelles in live cells and Hoechst (1 µg/mL) for fixed and live cell fluorescent staining of DNA and nuclei in cellular imaging technique. Cells were rinsed with fresh PBS and cellular fixation was performed with high purity paraformaldehyde (4% *w*/*v*) in PBS. For the straining of cytoskeleton actin, cells were permeabilized with 0.02% *w*/*v* Triton X-100 with 10% bovine serum albumin (BSA) and treated with a high-affinity F-actin probe, conjugated to green-fluorescent Alexa Fluor^®^ 488 dye (ActinGreen™ 488 ReadyProbes^®^ Reagent, TermoFisher, Waltham, Massachusetts, USA). LSM imaging was performed with an Olympus FV1000 confocal laser scanning microscope (Olympus, Shinjuku, Japan), equipped with diode UV (405 nm, 50 mW), multiline Argon (457, 488, 515 nm, total 30 mW), HeNe(G) (543 nm, 1 mW) and HeNe(R) (633 nm, 1 mW) lasers. An oil immersion objective (60xO PLAPO) and spectral filtering systems were used. The detector gain was fixed at a constant value and images were collected, in sequential mode randomly all through the area of the well. The image analysis was carried out using Huygens Essential software (by Scientific Volume Imaging B.V., The Netherlands). The statistical analysis was performed with ImageJ software and one-way ANOVA test.

## 5. Conclusions

In this work, core-shell nanosystems of metallic gold nanoparticles and hyaluronic acid were synthesized by a novel green one-pot approach based on a wet redox reaction at room temperature, thus tested as versatile anti-angiogenic platforms for tumor therapy applications. The HA-capped nanoparticles, prepared by using either 200 kDa (pro-angiogenic) or 700 kDa (anti-angiogenic) hyaluronan, were demonstrated stable against aggregation. The comparative analyses by UV visible spectroscopy, DLS and AFM pointed to a different grafting density of the biopolymer chains depending on the HA molecular weight, which in turn affected the plasmonic features (optical size), the hydrodynamic diameter and the nanoparticle stability. Antibacterial assays against Gram-negative *E. coli* and Gram-positive *S. aureus* demonstrated an enhanced inhibitory activity for the HA-capped AuNPs compared to the bare, glucose-capped AuNPs. Although the cell viability assays demonstrated an increased cytotoxicity in neuroblastoma than in prostate cancer cells upon the cellular treatments by HA-AuNP compared to the bare AuNP, the confocal imaging analyses unraveled a receptor-dependent perturbation effect on cytoskeleton actin as well as lysosomes.

Of note, in PC-3, a higher cellular uptake was detected for the hyaluronan-coated nanoparticles than the bare AuNPs. Moreover, significant differences were found in the cytoskeleton actin staining upon the treatments with the metal nanoparticles tailored by low- or high-molecular weight hyaluronan chains. These findings demonstrated the very promising potentialities of HA–AuNP systems for selective targeting and dose-dependent phototherapy-induced cytotoxicity in cancer therapy, with the possibility to modulate the anti-angiogenic effects by a controlled perturbation of cell migration processes in cancer metastasis.

## Figures and Tables

**Table 1:** Optical parameters (full width at half maximum, wavelength and absorbance at the maximum) from the UV-vis spectra of AuNP, bare and hyaluronic acid (HA)-conjugated samples. Averaged and standard deviation values from three repeated syntheses of each sample.

Sample	FWHM ± S.D. (nm)	λ_max_ ± S.D. (nm)	A_max_ ± S.D
AuNP	61 ± 4	531 ± 1	0.21 ± 0.04
HA(200)Au	88 ± 5	568 ± 1	0.32 ± 0.02
HA(700)Au	95 ± 3	555 ± 1	0.22 ± 0.02

**Table 2:** Optical parameters (full width at half maximum, wavelength and absorbance at the maximum) measured from the UV-vis spectra of AuNP, bare and HA-conjugated samples for the pellets ‘2’ (bare NPs) or pellets ‘3’ (HA-conjugated NP) immediately after resuspension in ultrapure MilliQ water (t = 0) and at increasing aging times (up to t = 10 days). The difference with respect to the freshly prepared pellets (t = 0) for FWHM and λ, as well as the relative percentage variation in Abs, are reported in brackets for comparison.

Sample	FWHM (ΔFWHM), nm	λ_max_ (Δλ_max_), nm	A_max_ (ΔA, %), nm
AuNP (t = 0)	57 (0)	535 (0)	0.567 (0)
AuNP (t = 2 days)	59 (2)	537 (2)	0.402 (50%)
AuNP (t = 7 days)	58 (1)	537 (2)	0.324 (29%)
AuNP (t = 8 days)	57 (0)	537 (2)	0.291 (49%)
AuNP (t = 10 days)	59 (2)	537 (2)	0.216 (62%)
HA(200)Au (t = 0)	82 (0)	561 (0)	0.554 (0)
HA(200)Au (t = 2 days)	81 (−1)	560 (−1)	0.486 (12)
HA(200)Au (t = 7 days)	94 (12)	566 (5)	0.345 (38)
HA(200)Au (t = 8 days)	92 (10)	566 (5)	0.294 (47)
HA(200)Au (t = 10 days)	107 (25)	566 (5)	0.231 (58)
HA(700)Au (t = 0)	84 (0)	548 (0)	0.394 (0)
HA(700)Au (t = 2 days)	86 (2)	549 (1)	0.347 (12%)
HA(700)Au (t = 7 days)	87 (3)	548 (0)	0.296 (25%)
HA(700)Au (t = 8 days)	93 (9)	549 (1)	0.223 (43%)
HA(700)Au (t = 10 days)	93 (9)	549 (1)	0.132 (66%)

**Table 3:** Nanoparticle size, molar extinction coefficient and concentrations for as-prepared and recovered pellets of AuNP and hybrid HA–AuNP at increasing aging times (up to t = 10 days).

Sample	d (nm)	ε (10^8^ mol^−1^·L·cm ^−1^)	*c* (10^−12^ mol·L^−1^)	NP/mL
AuNP_as prep_ (t = 0)	47	150	160	2.0 × 10^4^
AuNP (t = 0)	55	265	220	2.6 × 10^4^
AuNP (t = 2 days)	60	35.1	120	1.0 × 10^4^
AuNP (t = 7 days)	60	35.1	90	8.3 × 10^3^
AuNP (t = 8 days)	60	35.1	80	7.4 × 10^3^
AuNP (t = 10 days)	60	35.1	60	5.5 × 10^3^
HA(200)Au_as prep_ (t = 0)	148	2480	11	6.8 × 10^1^
HA(200)Au (t = 0)	126	1960	30	2.8 × 10^2^
HA(200)Au (t = 2 days)	126	1900	30	2.7 × 10^2^
HA(200)Au (t = 7 days)	140	2280	20	1.1 × 10^2^
HA(200)Au (t = 8 days)	140	2280	10	9.2 × 10^1^
HA(200)Au (t=10 days)	140	2280	10	7.2 × 10^1^
HA(700)Au_as prep_ (t = 0)	107	1540	11	2.0 × 10^2^
HA(700)Au (t = 0)	90	1200	30	8.7 × 10^2^
HA(700)Au (t = 2 days)	93	1250	30	6.7 × 10^2^
HA(700)Au (t = 7 days)	90	1200	20	6.5 × 10^2^
HA(700)Au (t = 8 days)	93	1250	20	4.3 × 10^2^
HA(700)Au (t=10 days)	93	1250	10	2.6 × 10^2^

**Table 4:** Inhibitory activity of bare and HA-capped AuNPs against *Escherichia coli* ATCC 9637 and *Staphylococcus aureus* ATCC 29213 in the comparison with the reference chloramphenicol (CHL).

Sample	*E. coli* ATCC 9637 or *S. aureus* ATCC 29213	
	Minimum Inhibitory Concentration (MIC)	
	nM	µg/mL
AuNP	0.58	1.4 × 10^−1^
HA(200)Au	0.55	1.4 × 10^−5^
HA(700)Au	0.42	1.0 × 10^−5^
CHL		8.0

## Abbreviations

AuNP	Gold Nanoparticles
CD44	Cluster Determinant 44
DLS	Dynamic Light Scattering
DMEM	Dulbecco’s Modified Eagle Medium
DMSO	Dimethyl sulfoxide
*E. coli*	*Escherichia coli*
EPR	Enhanced Permeability Retention
FBS	Foetal Bovine Serum
FWHM	Full Width at Half Maximum
HA	Hyaluronic Acid
HUVEC	Human Umbilical Vein Endothelial Cell
LSM	Laser Scanning Microscopy
MIC	Minimum Inhibitory Concentration
NPs	Nanoparticles
*S. aureus*	*Staphylococcus aureus*
S.E.M.	Standard Error of Means
VEGF	Vascular-Endothelial Growth Factor
